# Modulation of the Osteosarcoma Expression Phenotype by MicroRNAs

**DOI:** 10.1371/journal.pone.0048086

**Published:** 2012-10-25

**Authors:** Heidi M. Namløs, Leonardo A. Meza-Zepeda, Tale Barøy, Ingrid H. G. Østensen, Stine H. Kresse, Marieke L. Kuijjer, Massimo Serra, Horst Bürger, Anne-Marie Cleton-Jansen, Ola Myklebost

**Affiliations:** 1 The EuroBoNet Network of Excellence on Bone Tumours; 2 Department of Tumor Biology, Institute for Cancer Research, The Norwegian Radium Hospital, Oslo University Hospital, Oslo, Norway; 3 Norwegian Microarray Consortium, Department of Molecular Biosciences, University of Oslo, Oslo, Norway; 4 Department of Pathology, Leiden University Medical Centre, Leiden, The Netherlands; 5 Laboratory of Experimental Oncology, Rizzoli Orthopaedic Institute, Bologna, Italy; 6 Institute of Pathology, University of Münster, Münster, Germany; University of Chicago, United States of America

## Abstract

**Background:**

Osteosarcomas are the most common primary malignant tumors of bone and show multiple and complex genomic aberrations. miRNAs are non-coding RNAs capable of regulating gene expression at the post transcriptional level, and miRNAs and their target genes may represent novel therapeutic targets or biomarkers for osteosarcoma. In order to investigate the involvement of miRNAs in osteosarcoma development, global microarray analyses of a panel of 19 human osteosarcoma cell lines was performed.

**Principal findings:**

We identified 177 miRNAs that were differentially expressed in osteosarcoma cell lines relative to normal bone. Among these, miR-126/miR-126*, miR-142-3p, miR-150, miR-223, miR-486-5p and members of the miR-1/miR-133a, miR-144/miR-451, miR-195/miR-497 and miR-206/miR-133b clusters were found to be downregulated in osteosarcoma cell lines. All miRNAs in the paralogous clusters miR-17-92, miR-106b-25 and miR-106a-92 were overexpressed. Furthermore, the upregulated miRNAs included miR-9/miR-9*, miR-21*, miR-31/miR-31*, miR-196a/miR-196b, miR-374a and members of the miR-29 and miR-130/301 families. The most interesting inversely correlated miRNA/mRNA pairs in osteosarcoma cell lines included miR-9/TGFBR2 and miR-29/p85α regulatory subunit of PI3K. PTEN mRNA correlated inversely with miR-92a and members of the miR-17 and miR-130/301 families. Expression profiles of selected miRNAs were confirmed in clinical samples. A set of miRNAs, miR-1, miR-18a, miR-18b, miR-19b, miR-31, miR-126, miR-142-3p, miR-133b, miR-144, miR-195, miR-223, miR-451 and miR-497 was identified with an intermediate expression level in osteosarcoma clinical samples compared to osteoblasts and bone, which may reflect the differentiation level of osteosarcoma relative to the undifferentiated osteoblast and fully differentiated normal bone. Significance: This study provides an integrated analysis of miRNA and mRNA in osteosarcoma, and gives new insight into the complex genetic mechanisms of osteosarcoma development and progression.

## Introduction

MicroRNAs (miRNAs) are small, non-coding RNA molecules that are highly conserved across species and play key roles as regulators of gene expression. miRNAs have been estimated to regulate as much as 60% of the human protein coding genes [1], and modulate the levels of proteins involved in most biological processes, including development, cell proliferation, apoptosis and differentiation.

miRNAs are transcribed as monocistronic or polycistronic stem-loop RNA structures. The polycistronic transcripts contain clusters of several collinear immature miRNAs. After several steps of processing, a miRNA duplex of 20–22 nucleotides is formed from each individual hairpin-forming miRNA transcript. One of the strands of the hairpin loop is thought to be involved in gene regulation, whereas the other, the “passenger strand” or miRNA*, is expected to be less active and more frequently degraded. The active miRNA is preferentially incorporated into the RNA-induced silencing complex (RISC), which recognizes specific mRNA targets through complementary binding, mainly mediated through the “seed” sequence. miRNAs with identical seed sequences may target the same mRNA, and are grouped into miRNA families. On the other hand, the same miRNA may target multiple mRNAs. miRNA targeting causes posttranscriptional gene silencing by inducing mRNA degradation or repressing translation (reviewed in [2]).

Many studies have shown that miRNAs are aberrantly regulated in human cancers, suggesting a role as a novel class of oncogenes and tumor suppressors. miRNA expression profiles can distinguish tumors from corresponding normal tissues, as well as by their developmental origin and differentiation state [3], [4], [5].

Osteosarcoma is the most frequent primary malignant bone tumor in children and young adolescents. Survival rates have improved considerable after the introduction of multiagent chemotherapy in the 1980s, with a 5-year survival rate of 60–65% for patients without evidence of metastasis (reviewed in [6]). However, the survival rates have reached a plateau, and further improvements are probably dependent on novel biology-based therapies. At the molecular level, conventional osteosarcomas show complex genomic aberrations and highly variable patterns of gene expression [7]. miRNAs deregulated in human osteosarcoma compared to bone, osteoblasts and mesenchymal stem cells were recently published [8], [9], [10], [11], [12]. In addition, a few studies describing expression of selected miRNAs in osteosarcomas [13], [14], [15] are available, as well as a database of miRNA in sarcoma [16]. Using microarray profiling in an integrative approach, we have analysed genome-wide miRNA and mRNA expression patterns for the EuroBoNeT osteosarcoma cell line panel [17] compared against normal bone. Expression profiles of selected miRNAs were confirmed in clinical samples compared to bone and osteoblasts, providing new insight into the complex genetic mechanisms of osteosarcoma development and progression.

## Materials and Methods

### Ethic Statement

Use of patient samples was according to local medical ethical regulation for each EuroBoNet partner institute. For the Norwegian cohort, the information given to the patients, the written consent used, the collection of samples and the research project were approved by the Regional Ethical Committee for Southern Norway (Project #S-06133).

### Osteosarcoma and Normal Samples

The EuroBoNeT panel of human osteosarcoma cell lines [17] (n = 19) HAL, HOS, 143B, IOR/MOS, IOR/OS9, IOR/OS10, IOR/OS14, IOR/OS15, IOR/OS18, SARG, KPD, MG-63, MHM, MNNG/HOS, OHS, OSA, Saos-2, U-2 OS and ZK-58 were derived from ATCC (www.lgcstandards-atcc.org) or different partner laboratories within EuroBoNeT. The cell lines were grown as previously described [17]. Cell line authentication was performed by STR DNA profiling using Powerplex 16 (Promega, Madison, USA), and the data were validated using the profiles of the EuroBoNeT cell bank [17] and ATCC.

Primary clinical osteosarcoma samples were obtained from the Norwegian Radium Hospital (n = 12), and from a panel collected within EuroBoNeT (n = 71) ([18], [19]. Normal long bones were purchased from Capital Biosciences (n = 2) (Maryland, USA) or obtained from amputations of cancer patients at the Norwegian Radium Hospital (n = 4) and University College London (n = 1) where the normal samples were collected distant from the margin of the tumor.

Commercially available primary osteoblast cultures isolated from human calvaria (n = 2) (Sciencell Research Laboratories, California, USA) and from femur and tibia of different donors (n = 3) (Cambrex BioScience, Maryland, USA) were included. The osteoblast cells were maintained in medium provided by the manufacturer, split when reaching 80% confluency, and harvested when enough cells for DNA and RNA isolation were obtained.

An overview of the different cohorts used in the experiments is given in Table S1.

### RNA Isolation

Total RNA, including small RNAs, from osteosarcoma cell lines, clinical samples, bone and osteoblasts was extracted using the Qiagen miRNeasy Mini kit (Qiagen, GmbH, Hilden, Germany) according to the manufacturer’s protocol. Total RNA, not including small RNAs, from osteosarcoma cell lines, clinical samples and bone was extracted using TRIZOL (Invitrogen, California, USA) followed by RNA cleanup using the Qiagen RNeasy Mini kit (Qiagen) with on-column DNase treatment. RNA purity and quantity were measured on a NanoDrop ND-1000 spectrophotometer (Nanodrop Technologies, Delaware, USA), and RNA integrity was evaluated using an Agilent 2100 Bioanalyzer (Agilent Technologies Inc., California, USA).

### miRNA and mRNA Expression Profiling

miRNA expression profiling was performed using the Agilent miRNA Microarray System and the miRNA Complete Labeling and Hyb Kit Version 2.0, and hybridized to Agilent Human miRNA Microarrays (version 2, 799 human miRNAs) following the manufacturer’s instructions. miRNA data was imported into GeneSpring GX10 (Agilent), and the intensity values were log_2_ transformed and quantile normalized. miRNAs with detectable expression in at least 75% of the bone samples and/or 25% of the osteosarcoma cell lines were retained for further analysis, enabling the identification of miRNAs present in only a subgroup of the cell lines.

cDNA synthesis, cRNA amplification and Illumina Human-6 v2.0 Expression BeadChip hybridization for mRNA expression profiling were performed as previously described [18]. The data was extracted and quality controlled using the Gene Expression module v3.1.7 of Illuminás BeadStudio software (v3.1.0.0). Variance-stabilizing transformation [20] and quantile normalization on the probe level were carried out using the statistical package R [21] and the Bioconductor package lumi [22], [23].

MIAME (minimum information about a microarray experiment) compliant data can be downloaded from the GEO repository (www.ncbi.nlm.nih.gov/geo/), accession number GSE28425 for the miRNA and mRNA of the cell lines and bone and GSE30699 for the previously published mRNA data of the clinical samples [19].

### Quantitative Real-time Reverse-transcription PCR

Quantitative real-time reverse-transcription PCR (qRT-PCR) was performed using the ABI PRISM 7500 DNA Sequence Detection System (Life Technologies, California, USA). The TaqMan MicroRNA Reverse Transcription Kit with Megaplex Primer Pool was used to generate cDNA, and TaqMan MicroRNA Assays (Life Technologies) (Table S2) was used to quantitatively detect mature miRNAs. RNU44 and RNU6B were used as endogenous reference genes for normalization. The relative expression levels were determined using the comparative threshold cycle (2^−ΔΔCt^) method as described by the manufacturer. For miRNAs with undetectable expression levels in the clinical samples, the Ct-value was set to 40. miRNA qRT-PCR data of clinical samples, bone and osteoblasts were analyzed using Mann-Whitney U test with p-value cut-off of 0.05, and p-values between 0.05 and 0.15 were regarded as showing a trend towards significance.

### Analysis and Integration of miRNA and mRNA Data

Statistical tests were performed to identify miRNAs and mRNAs that were significantly differentially expressed between osteosarcoma cell lines or clinical samples and normal bone; the miRNA and mRNA genome-wide expression data were imported into GeneSpring GX10 (Agilent) and t-tests were performed applying a Benjamini and Hochberg FDR adjusted p-value cut-off of 0.05.

Predicted mRNA targets containing conserved binding sites for the miRNAs were extracted from the database TargetScan v5.1 (www.targetscan.org) using GeneSpring GX10. Targets for the miRNA* were predicted using TargetScan custom v5.1. Pearson’s correlation (*r*) was calculated for each miRNA and its predicted mRNA targets across all the osteosarcoma cell line samples. miRNA- mRNA pairs with *r* <−0.5 were selected for further analysis. Selected predicted targets were imported into MetaCoreTM (GeneGo, Michigan, USA) in order to identify pathways, biological functions and molecular interactions of the candidate target genes. The top significant enriched pathways and networks are presented, given a p-value significance level with a false discovery rate (FDR) multiple testing correction based on the Simes modification of the Bonferroni procedure [24].

## Results

### Global miRNA and mRNA Expression Patterns

**Figure 1:**
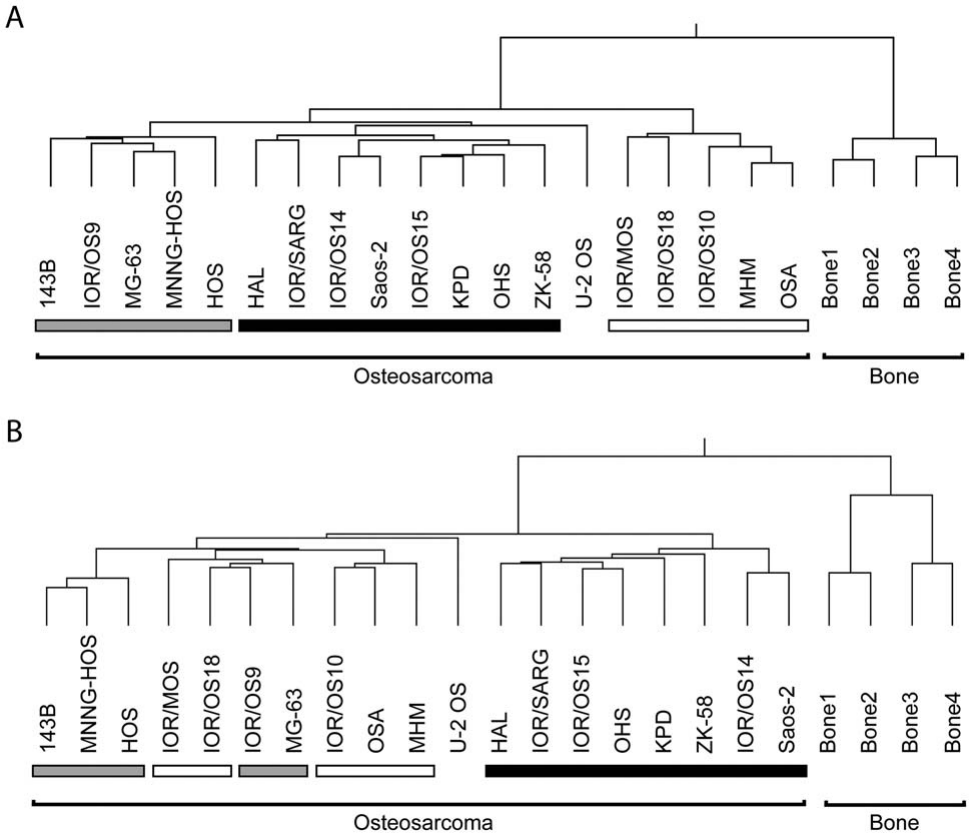
Unsupervised hierarchical clustering of 19 osteosarcoma (OS) cell lines and four normal bone samples. A . 361 miRNAs expressed in at least 75% of the bone samples and/or 25% of the osteosarcoma cell lines. **B**: Expression of the 48,701 mRNA probes. Distance metric: Pearson’s correlation absolute average. Bars under cell lines indicate samples with similar expression profile in miRNA (A) and mRNA (B) data.

After processing of the miRNA expression profiles of 19 EuroBoNeT human osteosarcoma cell lines and four bone samples, 340 miRNAs were identified as expressed in at least 25% of the cell lines and/or 75% of the normal bone samples. mRNA profiles were also obtained, and distinct miRNA or mRNA patterns could be identified that distinguished certain subclasses of the osteosarcoma cell lines, clearly distinct from the pattern of normal bone. The main class determined by miRNA pattern was also identified by mRNA pattern, indicated in black in Figure 1 whereas the two other subclasses were intermixed. U2OS separates for the main subclusters, but the difference in branch length is marginal.

### Deregulated miRNAs in Osteosarcoma Cell Lines

A statistical analysis identified 177 miRNAs, including 38 miRNA*s, that were significantly differently expressed between osteosarcoma cell lines and normal bone. All but 10 of the miRNAs had a more than two-fold change of intensity, 103 were upregulated and 74 were downregulated in the osteosarcoma cell lines compared to the mean expression level of the bones (Table S3). The top 10 up- and downregulated miRNAs are listed in bold in Table 1.

**Table 1:** Down- and upregulated miRNAs in osteosarcoma cell lines versus bone.

miRNA	miR family	miR cluster	Fold change	Presence CL/Bone
**Downregulated**				
**miR-451**		miR-451/miR-144	−28000	1/4
**miR-1**	miR-1/206	miR-1/miR-133a	−550	4/All
miR-144/miR-144*		miR-451/miR-144	−280/−87	0/3
**miR-223**			−240	0/All
**miR-142-3p**			−190	7/4
**miR-133b**	miR-133	miR-206/miR-133b	−86	4/All
**miR-150**			−84	0/4
**miR-206**	miR-1/206	miR-206/miR-133b	−81	0/All
miR-126/126*			−65/−30	17/4 (1/4)
**miR-486-5p**			−42	5/4
miR-195	miR-15/16/195/424/497	miR-195/miR-497	−24	All
miR-497	miR-15/16/195/424/497	miR-195/miR-497	−22	12/4
miR-363	miR-25/32/92/92ab/363/367		−14	6/All
miR-133a	miR-133	miR-1/miR-133a	−9.2	3/4
**Upregulated**				
**miR-18a**	miR-18	miR-17-92	95	All/2
miR-9/miR-9*			65/42	18/0
**miR-301a**	miR-130/301		47	All/2
**miR-18b**	miR-18	miR-106a-92	47	All/1
miR-31/miR-31*			35	14/1 (14/0)
**miR-503**			27	18/0
**miR-301b**	miR-130/301	miR-301b/130b	23	All/0
miR-21*			22	18/1
**miR-7**			21	18/1
**miR-137**			19	10/0
miR-96		miR-96/miR-182	18	All/2
miR-130b	miR-130/301	miR-301b/130b	14	All/1
miR-19a	miR-19	miR-17-92a	13	All
miR-196a	miR-196		10	All/2
miR-542-3p		miR-542/miR-450a	8.3	All/2
miR-29b	miR-29	miR-29b-2/miR-29c	7.1	All
miR-106a	miR-17-5p/20/93/106/519	miR-106a-92	7.1	All/2
miR-32	miR-25/32/92/92ab/363/367		6.5	All/1
miR-421		miR-421/374b	6.1	All/1
miR-17	miR-17-5p/20/93/106/519	miR-17-92a	5.8	All
miR-99b		miR-99b/let7/miR-125a	5.7	All
miR-20a	miR-17-5p/20/93/106/519	miR-17-92a	5.5	All
miR-125a-5p		miR-99b/let7/miR-125a	5.3	All
miR-93	miR-17-5p/20/93/106/519	miR-106b-25	4.9	All
miR-19b	miR-19	miR-17-92/miR-106a-92	4.8	All
miR-106b	miR-17-5p/20/93/106/519	miR-106b-25	4.5	All
miR-182		miR-96/miR-182	4.5	12/0
miR-450a		miR-542/miR-450a	4.4	All/1
miR-20b	miR-17-5p/20/93/106/519	miR-106a-92	4.3	All
miR-196b	miR-196		4.3	All
miR-374a	miR-374		4.0	All
let-7e		miR-99b/let7/miR-125a	3.8	All
miR-181b	miR-181		3.6	All
miR-374b	miR-374	miR-421/374b	3.3	All
miR-103	miR-103/107		2.9	All
miR-181d	miR-181		2.7	All/3
miR-29a	miR-29	miR-29a/miR-29b-1	2.5	All
miR-25	miR-25/32/92/92ab/363/367	miR-106b-25	2.4	All
miR-92a	miR-25/32/92/92ab/363/367	miR-17-92/miR-106a-92	2.3	All
miR-107	miR-103/107		2.1	All

The table lists 43 conserved miRNAs that are transcribed together from polycistronic miRNA clusters and/or belong to the same conserved families. In addition the top10 miRNAs are included (bold), of which some belong to the common families or clusters. miRNAs with common features are annotated with miRNA family and/or cluster members. For miRNAs detected as present in only a minority of the samples, the fold change (mean of cell lines vs bone) could be overestimated. Cell lines (CL), n = 19; bone, n = 4.

Of these 177 identified miRNAs, 90 belonged to evolutionarily conserved miRNA families as defined by Friedman et al. [1], indicating functional conservation and important roles. Further, among these, 43 miRNAs (50%) belonged to common conserved families or were transcribed together from polycistronic miRNA clusters. Deregulated miRNA clusters have been shown to be significantly overrepresented compared to single miRNAs in most investigated diseases [25]. The 43 miRNAs are included in Table 1 and annotated with miRNA family or cluster relation. These groups of miRNAs showed comparable up- or downregulation, and a concerted action could ensure a synergistic, redundant and more flexible means of regulation and have a higher potential to influence complex cell signalling networks.

The highly downregulated miRNAs presented in Table 1 were miR-126/miR-126*, miR-142-3p, miR-150, miR-223, miR-363, miR-486-5p and members of the miR-1/miR-133a, miR-206/miR-133b, miR-451/miR-144 and miR-497/miR-195 clusters. The miR-17-92 and the paralogous miR-106b-25 and miR-106a-92 clusters encode 12 mature miRNAs of which all were significantly overexpressed in the osteosarcoma cell lines. Furthermore, the overexpressed miRNAs included miR-7, miR-9/miR-9*, miR-21*, miR-31/miR-31*, miR-181, miR-196a/miR-196b, miR-503 and members of the miR-29 and miR-130/301 families (Table 1).

**Figure 2:**
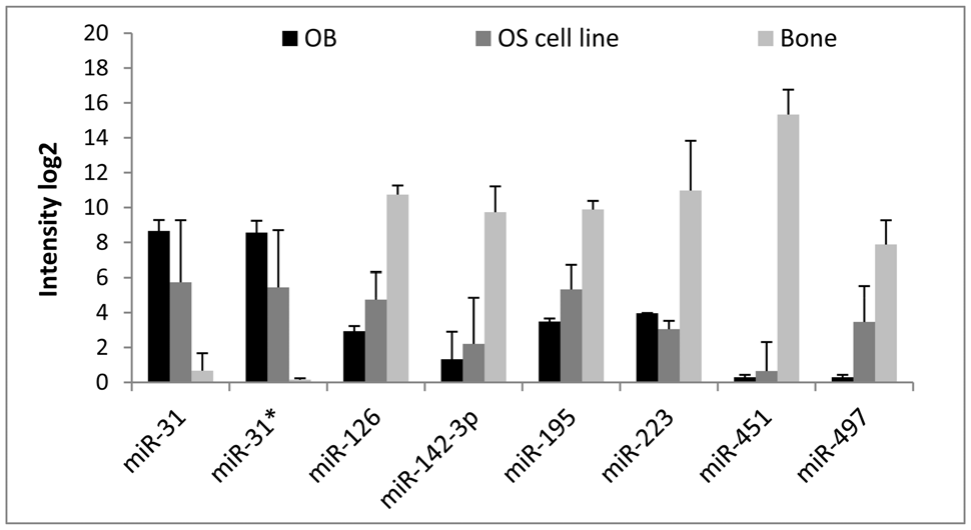
miRNA microarray expression levels in osteosarcoma cell lines, normal bone and osteoblasts. The expression level is shown as a mean for each group of samples; two osteoblast primary cultures, 19 osteosarcoma cell lines and four bones. OS: osteosarcoma; STDEV: standard deviation.

A global expression profile describing deregulated miRNAs in human osteosarcoma clinical samples compared to osteoblasts was recently published [8]. Among the list of 38 deregulated miRNAs identified by Maire et al., 16 miRNAs were also found to be deregulated in our cell lines and all but three miRNAs were found to be oppositely regulated in these two studies. Focusing on the conserved miRNAs presented in Table 1, we found that of the 14 miRNAs downregulated in our study relative to normal bone, six were published as upregulated in osteosarcoma relative to osteoblasts, namely the miRNAs miR-126, miR-142-3p, miR-195, miR-223, miR-451 and miR-497, while miR-31/miR-31* was upregulated compared to bone and downregulated compared to osteoblasts. To verify these findings, microarray expression data for osteoblasts were included for these miRNAs, and all, except for miR-223, showed the same expression pattern in our cell lines compared to osteoblasts as in the published clinical data (Figure 2).

### Validation of miRNA Expression in Clinical Samples

**Figure 3:**
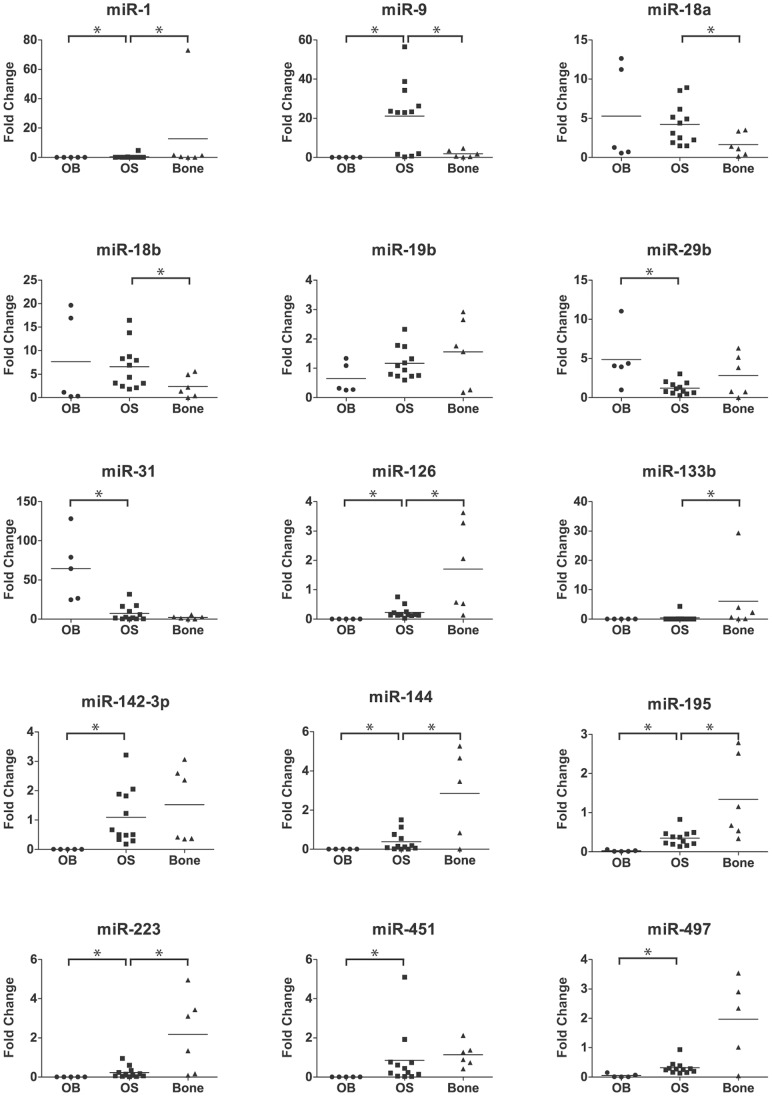
Verification of expression levels of miRNAs in osteosarcoma clinical samples, normal bone and osteoblasts. Scatter plot showing the expression level of 15 miRNAs, generated from qRT-PCR data. The expression values are relative to the mean expression level of bone samples. *: p-value <0.05; vertical line: mean value.

In order to verify the above findings in clinical samples, a smaller cohort of 12 clinical samples, six normal bone and five osteoblasts was collected. A subset of 15 miRNAs from Table 1 was selected for confirmation by qRT-PCR (Figure 3). Interestingly, all but two miRNAs showed an intermediate expression level in osteosarcoma clinical samples compared to the mean values in osteoblasts and bone, confirming the trend that was already observed in the cell lines. These 13 miRNAs include all the above seven miRNAs (omitting miR-31*) previously described in osteoblasts [8] as well as miR-1, miR-18a, miR-18b, miR-19b, miR-133b and miR-144.

Of the 15 miRNAs, all but miR-19b showed significant changes in gene expression level for clinical samples compared to either bone and/or osteoblasts. The expression pattern in cell lines compared to bone was confirmed in the clinical samples for 13 of 15 miRNAs. The level of change was significant for nine of these miRNAs; miR-1, miR-9, miR-18a, miR-18b, miR-126, miR-133b, miR-144, miR-195 and miR-223. miR-451 and miR-497 showed a trend towards being significantly decreased, miR-31 showed a heterogenous expression pattern, and miR-19b, miR-29b and miR-142-3p were expressed at comparable level in clinical samples and bone. These last miRNAs, miR-451, miR-31, miR-142-3p and miR-29b, as well as miR-1, miR-9, miR-126, miR-144, miR-195, miR-223 and miR-497, were all significantly different in clinical samples compared to osteoblasts. miR-133b showed a trend towards being significantly decreased, being undetected in two clinical samples and all osteoblasts. miR-144 was undetected in all osteoblasts, and miR-1 and miR-451 was undetected in two and three of the osteoblast samples, respectively.

### Expression of miR-17-92, miR-106b-25 and miR-106a-92 Clusters and Correlation with mRNA Host Genes

**Figure 4:**
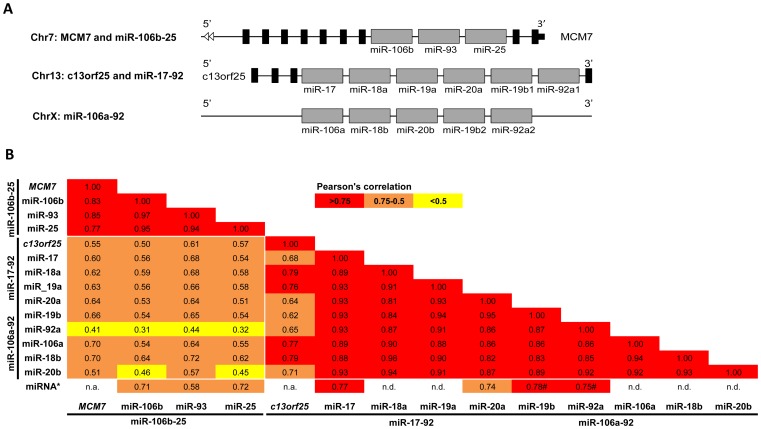
Genomic organization and Pearson’s correlation of miR-106b-25, miR-17-92, miR-106a-92 and mRNA host genes MCM7 and c13orf25. A. Schematic illustration of the genomic organization of miRNA clusters and host genes. *MCM7* and miR-106b-25 are located on 7q22.1, *c13orf25* and miR-17-92 are located on 13q31.2 and the miR-106a-92 cluster on Xq26.2. Black and grey boxes indicate exons and miRNAs, respectively, not in scale. B. Pearson’s correlation between the miRNAs of the miR-106b-25, miR-17-92 and miR-106a-92 clusters and the host genes *MCM7* and *c13orf25*. miR-17-92 and miR-106a-92 contains common mature miRNAs, hence the two clusters are not separated. miRNA* represents the correlation between the miRNA of the first strand and the second strand of the hairpin, ex between miR-17 and miR-17*. The values are colour-coded according to Pearson’s correlation in cell lines. #: Pearson’s correlation only calculated for miR-19b-1* and miR-92a-1* since miR-19b-2* and miR-92a-2* were not detected. n.d.: miRNA not detected in most of the samples due to low expression levels.

The miRNAs of the miR-17-92, miR-106b-25 and miR-106a-92 clusters had similar expression patterns in the osteosarcoma cell lines, although the miRNAs from the miR-106b-25 cluster showed slightly lower correlation with the other miRNA clusters (Figure 4). miR-18a of the miR-17-92 cluster and miR-18b of the miR-106a-92 cluster showed almost identical expression patterns in clinical samples, while some divergence was observed for miR-19b being encoded by both clusters (Figure 3). The more highly expressed miRNA*s in osteosarcoma cell lines showed high correlation with the miRNA of the complementary strand, as exemplified by miRNA/miRNA*s of the miR-106b-25 and miR-17-92 clusters (miRNA* in Figure 4B).

The miR-17-92 and miR-106b-25 clusters are encoded within introns of the mRNAs *c13orf25* and *MCM7*, respectively, and showed high positive correlation (*r* = 0.6–0.9) with the expression level of the host genes (Figure 4). *MCM7* and miR-106b-25, located at 7q22.1, were amplified in many of the cell lines (58%), while *c13orf25* and the miRNA cluster miR-17-92, located at 13q31.2, were amplified in only a minority of the cell lines and hemizygously deleted in 32% [26]. Despite the different patterns of amplification and deletions of these paralogous miRNA clusters, the expression levels of the miRNAs were strikingly similar. Thus, the copy number changes in the regions harboring these miRNAs seem to be overridden by other regulatory mechanisms.

The transcription factors E2F1, E2F2, E2F3 and MYC activate the members of the miR17-92 clusters [27], [28], [29], and MYC and E2F1 have also been shown to regulate the miR-106b-25 cluster [30]. *MYC*, *E2F1* and *E2F3* were amplified in a high number of the cell lines (74%, 68%, 47% and 42%, respectively) [26], and was also shown to be amplified in a set of 29 clinical samples (52%, 7%, 34% and 24%, respectively) [31]. However, no correlations (*r*<0.5) were observed between the expression levels of *E2F1*, *E2F2, E2F3* and *MYC* and the mature miRNAs of the three paralogous miRNA clusters miR-106b-25, miR-17-92 and miR-106a-92, neither at the RNA or protein level (not shown).

### mRNA Expression in Osteosarcoma Cell Lines and Clinical Samples

Statistical analysis identified 8,982 mRNA probes that were significantly differently expressed between the osteosarcoma cell lines and normal bone. About 50% of these genes (4,256 mRNAs) were confirmed to be changed also in clinical samples, being significantly differently expressed between 71 osteosarcoma clinical samples and normal bone. A hypergeometric test showed that the large overlap between the two datasets was highly significant with a p-value <1.0E–1000.

Analyses of functional annotation were performed on these gene sets using GeneGo, and the top 10 enriched processes in each of the sample categories were listed. Despite the observation that expression of numerous genes was unique to clinical or cell line samples, enrichment for similar processes like calcium signaling, apoptosis and cell differentiation was observed irrespective of sample origin (Table S4).

### Identification of Candidate miRNA Targets

An integrated analysis was performed on the miRNA and mRNA data to identify candidate miRNA targets. Several levels of filtering were applied to the lists of significantly differently expressed miRNAs and mRNAs. Firstly, predicted mRNA targets for the 90 conserved miRNAs were identified, as well as targets for the 29 miRNA*s encoded in the stem-loop sequence of miRNAs belonging to phylogenetically conserved families. These mRNA targets were filtered to only contain significantly differently expressed mRNA genes common to cell lines and clinical samples, thus avoiding *in vitro* artifacts. Secondly, the Pearson’s correlation for each of the miRNAs and its predicted targets across all the cell line samples was calculated. Pairs of miRNA or miRNA* and their predicted mRNA targets that did not show significant negative correlation (*r* >−0.5) were removed from the data set, resulting in a set of 199 pairs represented by 67 miRNA/miRNA* and 116 target mRNAs (Table S5). Analysis of functional annotation of these 116 mRNAs was performed, and the genes were found to be enriched in processes involved in apoptosis, calcium signaling and regulation of cell cycle, as well as pathways involving cytoskeleton remodeling, HIF regulation, PI3K pathway and development through IGF-1 and EGFR signaling (Table S4). Of the 15 miRNAs confirmed to be significantly differently expressed in clinical samples compared to either bone or osteoblasts, 8 miRNAs showed inverse correlation with at least one target mRNA, and these 30 mRNAs are highlighted in bold in Table S5.

The 116 mRNA targets were ranked based on the level of conservation of each of the miRNA target sites. A probability of preferentially conserved targeting (P_CT_) value has been calculated for all highly conserved miRNA families, where the P_CT_ value reflects the probability that a site is conserved due to selective maintenance of miRNA targeting rather than by chance or any other reason not pertinent to miRNA targeting [1]. miRNA/mRNA pairs with P_CT_ <0.4 were then removed from the dataset, but the miRNA*s were contained as no information regarding P_CT_ exists for these miRNAs, reducing the number of targets to 72 mRNAs. Twenty-six of the mRNAs showed an inverse correlation with at least two miRNAs each, mainly from the same miRNA family. These 80 pairs of 38 miRNA/miRNA*s and 26 mRNAs may identify the most interesting mRNA targets (Table 2).

**Table 2:** Potential target mRNAs of identified miRNAs.

Symbol	RefSeq ID	Name	miRNA
AKAP13	NM_006738.4	A kinase (PRKA) anchor protein 13	miR-17, miR-20a, miR-93, miR-106a, miR-106b
ARHGAP24	NM_001025616.1	Rho GTPase activating protein 24	miR-133a, miR-133b, miR-590-5p
CPEB3	NM_014912.3	cytoplasmic polyadenylation element binding protein 3	miR-29a, miR-29b
IRS2	NM_003749.2	insulin receptor substrate 2	?miR-7, miR-181b
KCNJ10	NM_002241.2	potassium inwardly-rectifying channel, subfamily J, member 10	miR-93, miR-106b
KIAA1632	NM_020964.1	KIAA1632	miR-25, miR-32
LIMK1	NM_016735.1	LIM domain kinase 1	miR-20a, miR-20b, miR-106a
MEX3B	NM_032246.3	mex-3 homolog B (C. elegans)	miR-29a, miR-29b
MPP1	NM_002436.2	membrane protein, palmitoylated 1, 55kDa	miR-32, miR-25
MTF1	NM_005955.1	metal-regulatory transcription factor 1	?miR-25, miR-130b
PIK3R1	NM_181504.2	phosphoinositide-3-kinase, regulatory subunit 1 (alpha)	miR-29a, miR-29b, miR-96
PIK3R3	NM_003629.2	phosphoinositide-3-kinase, regulatory subunit 3 (p55, gamma)	miR-21*, miR-29a, miR-29b
PTEN	NM_000314.3	phosphatase and tensin homolog (mutated in multiple advanced cancers 1)	miR-17, miR-20b, miR-9*, miR-92a
RHOC	NM_175744.3	ras homolog gene family, member C	miR-17, miR-20a, miR-20b, miR-106a
SAPS2	XM_942540.1	PREDICTED: SAPS domain family, member 2	miR-20b, miR-106a
SLC25A37	NM_016612.1	solute carrier family 25, member 37 (SLC25A37)	miR-181b, miR-181d
SLC2A3	NM_006931.1	solute carrier family 2 (facilitated glucose transporter), member 3	miR-195, miR-497
SNX4	NM_003794.2	sorting nexin 4	miR-29a, miR-29b
SOX5	NM_006940.4	SRY (sex determining region Y)-box 5	miR-195, miR-497, miR-503
SYT7	NM_004200.2	synaptotagmin VII	miR-17, miR-20a, miR-20b, miR-93, miR-106b
TGFBR2	NM_001024847.1	transforming growth factor, beta receptor II (70/80kDa)	miR-9, miR-590-5p
TNFRSF1B	NM_001066.2	tumor necrosis factor receptor superfamily, member 1B	miR-19a, miR-19b, miR-148b, miR-301a, miR-301b
WDR40A	NM_015397.1	WD repeat domain 40A	miR-29a, miR-29b
ZBTB47	NM_145166.2	zinc finger and BTB domain containing 47	miR-7-1*, miR-17, miR-18a, miR-18b, miR-19a, miR-19b, miR-20a, miR-20b, miR-106a
ZFP91	NM_170768.1	zinc finger protein 91 homolog (mouse)	?miR-17, miR-19a, miR-19b, ?miR-20a, ?miR-20b
ZNF385D	NM_024697.1	zinc finger protein 385D	miR-32, miR-92a

∧ miRNA with more than one binding site for the mRNA.

The mRNAs are differently expressed in osteosarcoma cell lines and clinical samples and predicted to be targeted by several of the differently expressed miRNAs. The pairs of miRNAs and mRNA show a Pearson’s correlation (r<−0.5) in cell lines.

Among these targets is transforming growth factor beta receptor II (*TGFBR2*), highly and significantly downregulated in both cell lines and clinical samples. The correlation plot of *TGFBR2* and miR-9 is shown in Figure S1. In the cell lines, miR-29a and miR-29b were inversely correlated with numerous interesting predicted targets like phosphoinositide-3-kinase regulatory subunit 1 (*PIK3R1/p85α*) and phosphoinositide-3-kinase regulatory subunit 3 (*PIK3R3/p55γ*), both central members of the PI3K/PTEN/Akt pathway. Insulin receptor substrate-1 (*IRS-1*), a downstream target of Akt, inversely correlated with miR-7 and miR-181b. In addition, the expression of the tumor suppressor gene phosphatase and tensin homolog (*PTEN*) inversely correlated with miR-17, miR-20b, miR-9* and miR-92a (Table 2), but also showed a modest inverse correlation (*r* = −0.4 to −0.5) with other miRNAs of the miR-17, miR-19, miR-130/301 and miR-26 families (Table S6).

## Discussion

Osteosarcomas show complex genomic changes with few recurrent chromosomal aberrations, which make it difficult to identify the molecular features that underlie the development of this type of cancer. As an increasing amount of high-throughput data from different genomic levels is being generated, the possibility of integrating these data gives us the unique opportunity to identify important genetic and epigenetic alterations in osteosarcoma. Among these, miRNAs and their target genes may represent potential novel therapeutic targets or biomarkers for osteosarcoma. In this study, we have identified deregulated miRNAs in osteosarcoma, and used the theoretical knowledge of predicted miRNA and mRNA interactions combined with experimental data to identify the most likely candidate mRNA targets and pathways.

Both miRNA and mRNA expression patterns clearly distinguished osteosarcoma cell lines from normal bone, as well as identified distinct subsets of samples. Since miRNA expression pattern might represent a certain cellular state and would impact on the mRNA transcriptome, one might expect that mRNA profiles are modulated by the miRNA patterns, so that subgroups defined by either would be overlapping. As shown, this was indeed the case, as the main osteosarcoma subcluster was identical whether based on miRNA or mRNA expression. Even though the other subclusters were less well maintained, as their branches were intermingled, this confirms the connection between miRNA patterns and the expressed transcriptome. No distinct phenotypic grouping according to previously identified properties of the cell lines was observed [17], [32]; rather these subsets reflect some other characteristic which is visible at both the miRNA and mRNA level.

A global expression profile describing the deregulation of 38 miRNAs in human osteosarcoma clinical samples compared to osteoblasts was recently published [8]. Of these, 16 miRNAs were also found to be deregulated in our cell panel. Interestingly, all but three miRNAs showed opposite regulation between these two studies, being upregulated in osteosarcoma clinical samples relative to osteoblasts and downregulated in our cell lines relative to normal bone and vice versa. To resolve these observations, seven of these miRNAs as well as eight additional deregulated and phylogenetic conserved miRNAs from the osteosarcoma cell line analysis were selected for validation in clinical samples. As predicted, the 13 miRNAs miR-1, miR-18a, miR-18b, miR-19b, miR-31, miR-126, miR-133b, miR-142-3p, miR-144, miR-195, miR-223, miR-451 and miR-497 showed opposite regulation when the osteosarcoma clinical samples were compared against bone or osteoblasts. The inverse deregulation of miRNAs compared to bone or osteoblasts is consistent with previous publications, Jones et al. [10] identified miR-126, miR-142-5p, miR-195, miR-223 and miR-451 to be downregulated in osteosarcoma versus bone while Lulla et al. [11] reported a subset of these, miR-126, miR-142-3p, miR-223 and miR-451, to be upregulated when compared to osteoblasts. Osteosarcoma has been regarded to develop as a result of genetic changes occurring during the determination or maturation of mesenchymal stem cells or committed progenitor cells (reviewed in [33], [34]). The miRNAs may play a role in regulating osteogenesis by the controlled temporal expression of different miRNA as differentiation proceeds, and thus the miRNA expression pattern observed in osteosarcoma may reflect a specific stage of differentiation. Similar modes of regulation have been described for miR-126/miR-126*, miR-223 and miR-451 in erythroid differentiation (reviewed in [34], [35]. Osteosarcomas express these miRNAs at an intermediate level compared to osteoblasts and bone, which may be consistent with the differentiation status of osteosarcoma relative to the undifferentiated osteoblasts and fully differentiated bones.

The deregulation of a number of miRNAs in human osteosarcoma compared to bone was recently reported. Thayanithy et al. [12]. and Jones et al. [10] reported 36 miRNAs and 34 miRNAs, respectively, to be deregulated in osteosarcoma clinical samples when compared to bone. The overlap between these two studies was marginal as only the downregulation of miR-150 was observed in both datasets. miR-150 acts as a tumor suppressor in malignant lymphoma through activation of the PI3K-Akt pathway [36]. Using a less stringent cut-off, we identified 177 deregulated miRNAs in osteosarcoma cell lines, of which 17/36 miRNAs and 16/34 miRNAs from the above studies were confirmed to be significantly changed, including the common miR-150. All but three of these miRNAs showed similar up/down-regulation as previously reported, further supporting our findings.

Previous studies have reported deregulation of specific miRNAs in osteosarcomas (reviewed in [37]). miR-143, down-regulated in osteosarcoma cell lines and primary tumor samples, promotes apoptosis, suppresses tumorigenicity [14] and regulates metastasis [13]. However, the downregulation of miR-143 in our osteosarcoma cell lines was not significant. miR-21 has been reported as overexpressed in a range of different tumors, including osteosarcoma tissue where it is involved in cell invasion and migration [15]. miR-21 was highly expressed in both our tumor and normal samples, whereas miR-21* was among the top 10 overexpressed miRNAs in our tumors.

Expression profiles of selected miRNAs were validated in clinical samples, both to avoid possible artefacts that may have arisen during *in vitro* culturing, or due to regulation reflecting the *in vitro* growth conditions. Confirming our *in vitro* findings, 13/15 miRNAs showed the same expression pattern in clinical samples compared to bone. The only exceptions were miR-19b and miR-29b, upregulated in cell lines while a not significant downregulation was observed in clinical samples. Downregulation of miR-29b in osteosarcoma compared to bone is consistent with a recent report [10]. The level of change was significant for nine of these 13 miRNAs. All of the miRNAs that were confirmed downregulated in clinical samples compared to bone are known to act as tumor suppressors in other types of cancers, that is miR-1, miR-126/miR-126*, miR-133b, miR-144, miR-195, miR-223 and miR-497 [38], [39], [40], [41], [42], [43].

miR-133b was expressed at low or undetectable level in most of the clinical samples, and was the strongest downregulated miRNA compared to bone. miR-133b was not detected in the osteoblasts. miR-133a/miR-133b have a dual role being essential for myogenesis and suppressing osteogenesis through targeting of runt-related transcription factor 2 (*RUNX2)*, and are downregulated in BMP2-induced osteogenesis of premyoblast mesenchymal cells [44]. RUNX2 is a master regulator of osteogenic differentiation, and the expression level increases gradually during osteogenesis with highest levels observed in early osteoblasts, but then decreases to very low levels in mature osteocytes [45]. Accordingly, *RUNX2* was significantly higher expressed in our osteosarcoma clinical samples, relative to normal bone (results not shown) as has been shown for the comparison of osteosarcoma tumors with osteoblasts [46]. RUNX2 expression has also been detected in most osteosarcoma primary tumors [47]. *RUNX2* does not seem to be reduced at the mRNA level by miR-133 in our study, which may be explained by the high amplification frequency (68%) of *RUNX2* observed in our cell lines, as previously reported [46].

All the miRNAs of the miR-17-92 cluster (13q31.2) and the paralogous miR-106b-25 (7q22.1) and miR-106a-92 clusters (Xq26.2) were highly expressed in osteosarcoma cell lines compared to normal bone, and all showed a similar expression pattern. The upregulation of miR-18a and miR-18b were verified in clinical samples. High levels and amplification of the miR-17-92 and miR-106b-25 clusters have been reported for a multitude of different cancers [5], [30], [48]. In two recent publications, miR-17-92 [9], [12] and miR-106a and miR-106b from the miR-106a-92 and miR-106b-25 clusters [9] were reported upregulated in osteosarcomas. In addition, haploinsuffiency of the miR-17-92 cluster is responsible for developmental defects in individuals with skeletal abnormalities, being the first example where a germline mutation of a miRNA cause hereditary disease [49].

Despite previous reports of amplification of the chromosomal regions containing the miR-106b-25 and miR-17-92, copy number changes were infrequent for these regions in osteosarcoma, strongly indicating that the expression levels are not driven by amplification. As one might expect, there was clear covariation of the host genes *MCM7* and *c13orf25* and their respective intronic miRNA clusters miR-106b-25 and miR-17-92, thus a common regulatory mechanism for both the miRNAs and their host genes seems likely, plausibly due to co-transcription from the mRNA promoter. It was recently shown that the majority of co-expressed miRNAs were under coordinated control by common transcription factors, and were more likely to be functionally related [50]. Previous reports have shown that the transcription factors MYC, E2F1, E2F2 and E2F3 activate the miR-17-92 cluster, and E2F1 has also been shown to activate the miR-106b-25 cluster (reviewed in [51]). E2F1-3 and MYC were frequently amplified in osteosarcoma cell lines and clinical samples [26], [31], but no correlation could be observed between the paralogous miRNAs and RNA or protein levels of E2F1-3 and MYC. However, a regulatory network was recently described in osteosarcoma, demonstrating that miRNAs of the 14q32 locus act cooperatively to destabilize MYC and thus control the expression of the miR-17-92 cluster [12].

A number of target genes regulated by the miR-106b-25, miR-17-92 and miR-106a-92 clusters have been reported, such as E2F1-3, cyclin-dependent kinase inhibitor (CDKN1A/p21), BCL2-like 11 (BCL2L11/Bim), RB1 and components of the TGF-β pathway [5], [30], [52]. miR-9 was highly expressed in osteosarcoma clinical samples and cell lines relative to normal tissue as has been observed for breast cancer [53]. In osteosarcoma cell lines, miR-9 inversely correlated with the predicted target gene transforming growth factor, beta receptor II (*TGFBR2*). An increase of miR-9 and reduced levels of *TGFBR2* was confirmed in osteosarcoma clinical samples. In line with this, overexpression of miR-106b or miR-93, as observed for osteosarcoma, renders gastric cancer cells insensitive to TGFB-mediated cell cycle arrest [30], consistent with an oncogenic role of the miR-106b-25 cluster.

Strong inverse correlation was observed between the tumor suppressor *PTEN* and several members of the miR-17, miR-19, miR-130/301 and miR-26 families, which were upregulated in the osteosarcoma cell lines. *PTEN* was downregulated in both osteosarcoma cell lines and clinical samples, and is a verified target of miR-26a and members of the miR-106b-25 and miR-17-92 clusters [54], [55], [56], [57]. PTEN antagonizes signaling through the PI3K/PTEN/Akt pathway, which plays a crucial role in tumorigenesis by promoting cell proliferation and inhibiting apoptosis. This pathway may also be affected by other osteosarcoma miRNAs, as the p85α regulatory subunit of PI3K showed strong inverse correlation with miR-29a and miR-29b in the cell lines. p85α is a verified target of the miR-29 family [58]. miR-29b was downregulated, although not at a significant level, in osteosarcoma clinical samples compared to bone, consistent with previous reports [10]. A positive correlation has been observed between expression of PTEN and the degree of differentiation in osteosarcoma specimens, and higher expression of PTEN was observed in benign lesions of bone than in osteosarcoma [59]. miRNAs are regarded as fine-tuners of gene expression, and slight variations in the amount of PTEN may contribute to the development of cancers (reviewed in [60]).

### Conclusions

A high number of deregulated miRNAs were identified in osteosarcoma, many of them known to have a role as tumor suppressors or oncogenes in other cancers. A number of identified miRNAs showed opposite regulation when compared to bone or osteoblasts. This observation suggests that osteosarcomas may represent a partially differentiated cell between the undifferentiated osteoblasts and a fully differentiated bone. Deregulated mRNA targets and pathways, like members of the PI3K/PTEN/Akt and TGFB pathway, are involved in important functions related to tumorigenicity, and play an important role in the development of osteosarcoma.

## Supporting Information

Figure S1 **Inverse correlation between** ***TGFBR2*** **and miR-9 in cell lines and normal bones.**
(PDF)

Table S1 **Overview of cell lines, clinical samples and normal bone used in the study.**
(XLS)

Table S2 **Overview of TaqMan assays for quantitative real-time PCR experiments.**
(XLS)

Table S3 **Overview of 177 miRNAs significantly differently expressed between osteosarcoma cell lines and bones, p≤0.05.**
(XLS)

Table S4 **GeneGO enrichment of pathway maps for functional annotations of gene sets unique to cell line, gene set common for cell lines and clinical samples as well as gene set of 116 genes predicted to be targets of miRNAs in osteosarcoma.**
(XLS)

Table S5 **The Pearson’s correlation (r) for each of the 67 miRNAs and its predicted mRNA targets across all the cell line samples.**
(XLS)

Table S6 **Pearson’s correlation (** ***r*** **) between miRNAs and the putative target gene** ***PTEN*** **.**
(XLS)
