# Supplementary material for: Modulation of the Osteosarcoma Expression Phenotype by MicroRNAs
Source: PLoS One. 2012 Oct 25;7(10):e48086. doi: 10.1371/journal.pone.0048086 (PMC3485010; doi:10.1371/journal.pone.0048086)

Supporting information Figure S1

Inverse correlation between *TGFBR2* and miR-9 in cell lines and normal bone.

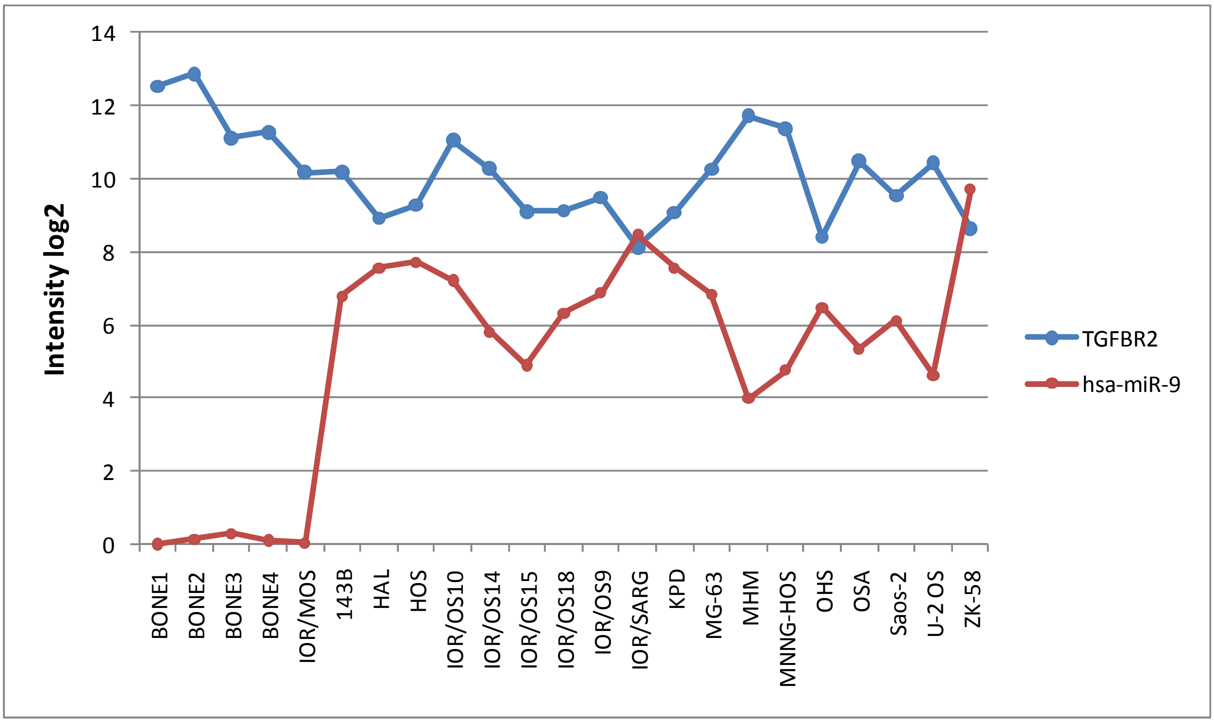

Supplement: Figure S1 — Inverse correlation between TGFBR2 and miR-9 in cell lines and normal bones. (PDF) [file pone.0048086.s001.pdf]
